# Effect of a Diet Supplemented with the *Moringa oleifera* Seed Powder on the Performance, Egg Quality, and Gene Expression in Japanese Laying Quail under Heat-Stress

**DOI:** 10.3390/ani10050809

**Published:** 2020-05-07

**Authors:** Reham Abou-Elkhair, Heba Abdo Basha, Walaa Slouma Hamouda Abd El Naby, Jamaan S. Ajarem, Saleh N. Maodaa, Ahmed A. Allam, Mohammed A. E. Naiel

**Affiliations:** 1 Nutrition and Clinical Nutrition Department, Faculty of Veterinary Medicine, University of Sadat City, Al Buhayrah, Menofia 32958, Egypt; 2 Poultry Breeding and Production in Department of Animal Husbandry and Animal Wealth Development, Faculty of Veterinary Medicine, Alexandria University, Alexandria 21500, Egypt; heba.basha@alexu.edu.eg; 3 Genetics and Genetic Engineering in Department of Animal Husbandry and Animal Wealth Development, Faculty of Veterinary Medicine, Alexandria University, Alexandria 21500, Egypt; walaa.hamouda@alexu.edu.eg; 4 Department of Zoology, College of Science, King Saud University, P.O. Box 2455, Riyadh 11451, Saudi Arabia; jajarem@ksu.edu.sa (J.S.A.); maodaa_28@yahoo.com (S.N.M.); 5 Department of Zoology, Faculty of Science, Beni-suef University, Beni-suef 65211, Egypt; allam1081981@yahoo.com; 6 Department of Animal Production, Faculty of Agriculture, Zagazig University, Zagazig 44511, Egypt

**Keywords:** *Moringa oleifera*, gene expression, reproductive, heat stress, quail

## Abstract

**Simple Summary:**

The debilitating effect of heat stress on egg production is well known, but its mechanism is not understood. Some studies attribute for this bad effect on the reduction in feed intake to high temperature or the relation between heat stress and reproductive and ovarian dysfunction, which cause a lowering in egg production. Heat stress directly affects ovarian function by altering the secretion of ovarian hormones such as prolactin. *Moringa* seed powder plays an effective role in overcoming the effect of heat stress, through the flavonoids and other phenolic compounds present in it. Hence, we investigated the effect of feeding *Moringa oleifera* seed powder with different concentrations, on egg production, egg quality trait, and the expression levels of selected ovarian genes in quail layers subject to heat stress. We found that supplementing laying Japanese quail feed with the *Moringa oleifera* seed powder at level 0.3% enhanced resistance to heat stress condition and consequently improved egg productivity.

**Abstract:**

This study was conducted to evaluate the effect of three concentrations of the *Moringa oleifera* seed powder as a feed supplement on the productive performance and egg quality traits of laying Japanese quail (*Coturnix japonica*) exposed to heat stress. The expression patterns of the genes estrogen receptors (*ESR2*), follicle-stimulating hormone receptor (*FSHR*), prolactin receptor (*PRLR*), and steroidogenic acute regulatory protein (*STAR*) were estimated in ovaries, using a quantitative real-time polymerase chain reaction. A total of 200 laying quail aged seven weeks were randomly allocated to the following four experimental groups—the control (CNT), T1, T2, and T3 groups; each group comprised 50 quail females with 5 replicates (10 per group). The CNT group was fed a basal diet, whereas the T1, T2, and T3 groups were fed the basal diet supplemented with 0.1%, 0.2%, and 0.3% *M. oleifera* seed powder, respectively. The results revealed that the T3 group showed the highest hen-day egg production (%) as well as the highest egg yolk index. Feed intake and feed conversion ratio improved significantly (*p* < 0.05) with increased concentrations of the *M. oleifera* seed powder supplementation. Furthermore, the mRNA expressions of *ESR2*, *FSHR*, and *STAR* increased significantly in the T3 group, compared to those in the CNT group. Alterations in ovarian gene expressions corresponded to the reproductive patterns of the treated Japanese quail. Thus, it was concluded that the supplementation of the Japanese quail feed with 0.3% *M. oleifera* seed powder during the laying period might enhance resistance to heat stress and consequently improve egg productivity.

## 1. Introduction

Eggs are considered to be one of the most valuable daily foods, such that the poultry egg industry has been intensively growing to meet the increased demand for eggs. Quail eggs can be used besides chicken eggs to meet this demand, and despite their small size, they exhibit several nutritional benefits because they are richer in protein, fat, vitamins, and minerals ( iron, potassium, and zinc) than chicken eggs [1,2]. In addition, it has been claimed that the eggs from Japanese quail are rich sources of protein with low fat and cholesterol [3]. Thus, many people, especially in Asian countries, consume quail eggs because they are a good source of nutrients for human health and help in the treatment of tuberculosis, bronchial asthma, and diabetes diseases [4].

Even at present, global warming has been affecting the poultry industry, in that high environmental temperatures impose severe stress on laying birds, thus, resulting in reduced egg production and increased economic loss [5]. The adverse effect of heat stress results in a reduced feed intake [6,7] as well as a reduced egg production [8], by modifying neuroendocrine activity by stimulating the hypothalamic–pituitary axis. Furthermore, heat stress negatively affects reproductive activity and ovarian function, eventually reducing egg production [8]. Heat stress directly affects ovarian function by altering folliculogenesis and the secretion of ovarian hormones, such as prolactin, luteinizing hormone (LH), and follicle-stimulating hormone (FSH) [9]. Ovarian functions and folliculogenesis are mainly controlled by various genes, such as steroidogenic acute regulatory protein (*STAR*), inhibin subunit alpha (*INHA*), bone morphogenetic protein 15 (*BMP-15*), follicle-stimulating hormone receptor (*FSHR*), and estrogen receptors 1 and 2 (*ESR1* and *ESR2*) [10,11,12,13,14].

Several studies have examined technical options such as nutritional manipulation and feed additives to alleviate the harmful effect of heat stress on laying birds [7,15,16,17]. Natural herbal plants, such as black seed, coriander seeds, and *Moringa oleifera* exhibit effective roles in overcoming the effect of heat stress through flavonoids and other phenolic compounds [18,19]. *M. oleifera* has a high nutritional value as it comprises significant concentrations of vitamins A, B, and C, proteins, phosphorus, and calcium [20,21]. Moreover, it is rich in flavonoids, carotenoids, tocopherols, selenium, and vitamin E [22,23] and has a potent antioxidant activity [24,25]. In addition, Ashour et al. [26] reported significantly increased egg production and improved hatchability, along with some egg quality parameters, and also lowered some blood biochemical components in Japanese quail that were fed an *M. oleifera* seeds-supplemented diet. Additionally, Lan et al. [27] revealed that supplementation of *Moringa* leaf powder at 5% had no effect on the performance of laying quail but remarkably enhanced yolk color. It is well-known that heat stress promotes oxidative stress in body cells, thereby, interrupting ovarian functions. In this context, the present study aimed to investigate the effects of feeding different concentrations of the *M. oleifera* seed powder on egg production, egg quality traits, and selected ovarian gene expressions in laying quail that were subject to heat stress.

## 2. Materials and Methods

All procedures by this study were in accordance with international ethical standards. The research involved no human participants. All animal procedure was performed following the guidelines for care and use of laboratory animals of the International Council for Laboratory Animals (ICLAS-2015). All procedures and experiments were accepted and approved by the local ethics committee of animal use, the Institutional Animal Care and Use Committee (AU-IACUC), Faculty of Veterinary Medicine, Alexandria University.

### 2.1. Animals, Housing, and Experimental Design

A total of 200 seven-week-old laying quail (*Coturnix japonica*) with an initial live weight of 140 ± 5 g was allocated to four groups—the CNT, T1, T2, and T3 groups; each group comprised 50 quail with 5 replicates (10 per group). The CNT group was administered a corn-soybean basal diet in a mash form without any additives. The basal diet was formulated to meet the nutritional requirements, according to the recommendation of the National Research Council (NRC) [24]. *M. oleifera* seed powder was obtained from the Agricultural Research Centre, Giza, Egypt (see Table 1). The ingredients, nutrient concentration, and chemical composition [28] of the basal diet are shown in Table 2. The T1, T2, and T3 groups were administered the same basal diet supplemented with 0.1%, 0.2% and 0.3%. *M. oleifera* seed powder, respectively. Approximately 30 ± 5 g per bird of food and water were available at all times during the experiment. During the pre-laying period, the quail were fed a commercial grower diet. The experiment was initiated at 7 weeks of age and completed at 15 weeks of age. The lighting regimen was 16 h of continuous light per day. All groups were exposed to heat stress (35 ± 1 °C) throughout the experimental period, using an electrical heater.

### 2.2. Productive and Reproductive Measurements 

Eggs were collected daily, and hen-day egg production (%) was calculated. Egg weight and feed consumption were recorded on five replicates, at weekly intervals. Feed conversion ratio (FCR) was estimated as the ratio of feed consumed (g)/egg weight (g).

### 2.3. Sample Collection and Egg Quality Measurements

Twenty-five eggs were randomly collected from quail in each treatment group (5 eggs/replicate), at 11 and 15 weeks of age, to evaluate the egg quality parameters. Shell thickness (without the inner and outer shell membranes) was measured at the blunt, equatorial, and sharp regions to obtain an average value [29].

Egg components, including the shell, albumen, and yolk, were weighed separately and expressed as percentages. The egg shape index was calculated according to the method described in [27]. Egg yolk index was calculated according to the method described by Romanoff and Romanoff [30]. The Haugh unit was estimated according to the method reported by Cotta [31].

At the end of the experiment when the quail were 15 weeks of age, 25 quail from each treatment group (5 birds/replicate) were randomly selected and slaughtered, and the whole ovaries were quickly removed and weighed. The tissue samples were homogenized, immediately snap-frozen in liquid nitrogen, and stored at −80 °C, until further use.

### 2.4. Total RNA Isolation and cDNA Synthesis

Total RNA was isolated from the ovaries using the Biozol reagent (Bioflux, Japan), according to the manufacturer’s instructions. cDNA was synthesized using the Sensi FAST™cDNA Synthesis Kit (Bioline, United Kingdom), according to the manufacturer’s instructions. In brief, 4 µL total RNA was mixed with 4 µL 5× Trans Amp buffer, 1 µL reverse transcriptase, and 11 µL RNase/DNase-free H_2_O. The thermal cycler program was conducted as follows—25 °C for 10 min, 42 °C for 15 min (reverse transcription), and 4 °C hold. The integrity of the cDNA was determined by amplification of ß-actin, and the sample was then stored at −20 °C until further use. 

### 2.5. Quantitative Real-Time Polymerase Chain Reaction (PCR)

Quantitative real-time PCR was conducted to reveal the expression profiles of *ESR2*, *FSHR*, *STAR*, and *PRLR*, as described previously by Abd El Naby and Basha [32]. Primer sequences and annealing temperatures for the real-time PCR are listed in Table 3. The housekeeping gene *β-actin* was used as the normalization control. Relative mRNA expression was calculated using the 2^(−△△ct)^ method, and the results were reported as a fold change [33].

### 2.6. Statistical Analysis

Experimental data were analyzed through one-way ANOVA, using the SAS (Statistical Analysis System, version 6, 4^th^ Edition, SAS Institute, Cary, NC, USA). Prior to ANOVA analysis, all data were tested for normality using the Shapiro-Wilk test [34]. Data are expressed as mean ± standard error of the mean, and significance was considered at *p* < 0.05. Analyses of significant treatment and replicate effects were performed using multiple range comparisons with Duncan’s multiple range test [35]. The quantitative real-time PCR data obtained were analyzed using the GraphPad Prism software version 6 (GraphPad Prism Software, La Jolla, CA, USA), through statistical analysis of one-way analysis of variance (ANOVA). 

## 3. Results

### 3.1. Productive and Laying Performance

The effects of different concentrations of the *Moringa oleifera* seed powder as a feed additive on the productive and laying performance of quail are summarized in Table 4. In the first 4 weeks of the experiment, no significant differences were observed in egg weight and feed intake among the treatment groups. In the next 4 weeks of the experiment, the administration of the *Moringa oleifera* seed powder increased the egg weight in descending order; T2, T3, and T1 groups, but decreased feed intake. The T2 and T3 groups showed the highest egg weight and the lowest feed intake during 11–15 weeks of age. Generally, laying quail fed a diet supplemented with *Moringa oleifera* seed powder had better feed conversion ratio and egg production % (*p* < 0.01) during the period 7–15^th^ weeks of age than the control birds. The T3 group, which received the highest concentration (0.3%) of the *Moringa oleifera* seed powder, had the lowest FCR and the highest egg production (%), as compared to the other treated groups.

### 3.2. Egg Quality Measurements

Data regarding egg quality parameters at the end of the 4th and 8th weeks of the experiment are presented in Table 5. No significant differences were observed in egg quality traits—shell thickness, albumen (%), yolk (%), egg yolk index, egg shape index, and the Haugh unit between the CNT and the TI, T2, and T3 groups at the end of 4th week of the experiment. Furthermore, the supplementation of *M. oleifera* seed powder in the diet did not affect the egg shape index and the Haugh unit at the end of the 8th week of the experiment. However, at the same age, the laying quail which received *M. oleifera* seed powder as a supplement had a higher shell thickness, albumen (%), and yolk (%) (*p* < 0.01) than those who received the basal diet alone. The eggs of the quail in the T3 group recorded the highest significant egg yolk index.

### 3.3. Expression Profiles of ESR2, FSHR, STAR, and PRLR

**Figure 1:**
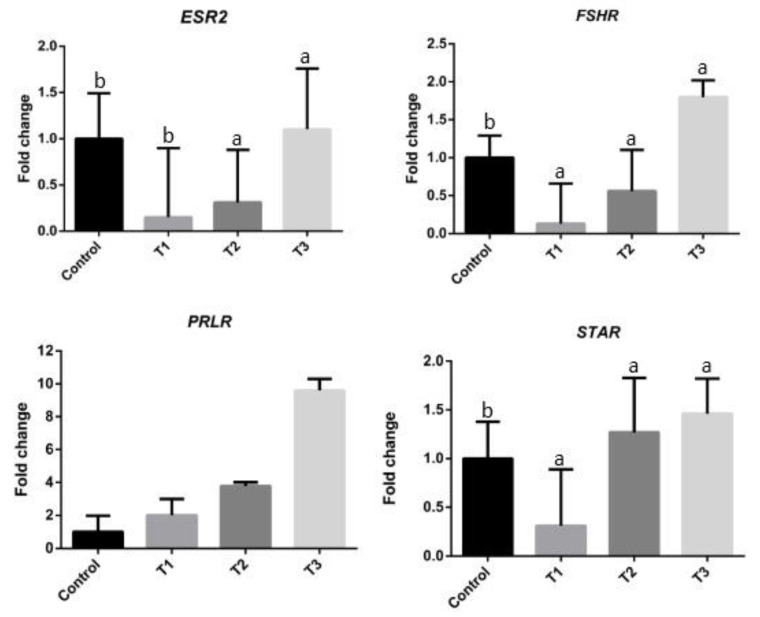
The relative expression levels of ovarian genes (*ESR2* = estrogen receptor 2 (ER beta)*, FSHR* = follicle-stimulating hormone receptor, *PRLR* = prolactin receptor, and *STAR* = steroidogenic acute regulatory protein) in quail, with different treatments. Letters a, and b on the data bars indicate when *p* > 0.05 significant. CNT (control), T1 (basal diet supplemented with 0.1% *Moringa oleifera* seed powder), T2 (basal diet supplemented with 0.2% *Moringa oleifera* seeds powder), and T3 (basal diet supplemented with and 0.3 % *Moringa oleifera* seeds powder).

The results of the expressions of the selected ovarian genes *ESR2*, *FSHR*, *STAR*, and *PRLR* are illustrated in Figure 1. The mRNA expressions of *ESR2*, *FSHR*, and *STAR* in the ovaries, increased significantly (*p* < 0.05) with increasing *Moringa oleifera* seed powder concentrations in the feed rations (T3 group), compared to the CNT group. However, the *FSHR* and *STAR* mRNA expressions showed significant decrease in T1 (*p* < 0.05) group compared to CNT group. The mRNA expressions of *FSHR* and *ESR2* were significantly decreased in T2 (*p* < 0.05) group compared to the CNT. Finally, the mRNA expression of *PRLR* was increased in the T3, T2, and T1 groups compared to the CNT group, with the T3 group showing the highest expression. 

## 4. Discussion

Excessive environmental heat is considered to be one of the major problems for laying birds. *M. oleifera* has potential as a valuable feed additive in animal nutrition, as it is rich in flavonoids and carotenoids, both of which are strong natural antioxidants [36]. It effectively prevents cellular oxidative damage by enhancing antioxidant enzyme activity, reducing free radicals, and decreasing lipid peroxidation [25]. The present study demonstrated the beneficial effects of the *M. oleifera* seed powder as a feed additive, in terms of alleviating the negative effects of heat stress on the laying, productive performance, and egg quality traits of Japanese quail. The feed intake of quail was significantly reduced in the T2 and T3 groups between 11 and 15 weeks of age, during the experiment. Furthermore, FCR was significantly higher in all supplemented groups (i.e., the T1, T2, and T3 groups) than in the CNT group, during the whole experiment. Hassan et al. [37] reported a higher FCR in broiler chickens reared under heat stress and fed a basal diet supplemented with 0.2% and 0.3% *M. oleifera* leaves for 35 days. Furthermore, Ahmad et al. [38] reported improvements in FCR/dozen eggs and egg weight in laying hens administered a diet supplemented with 5 g/kg *M. oleifera* pod meal. Additionally, David et al. [39] observed that replacing antibiotic growth promoters in broiler diet with herbal supplements of 0.1% and 0.05% Moringa leaf powder had beneficial effects on the growth performance.

The adverse effect of heat stress on egg production is well recognized, but its mechanism of action remains unclear. Changes in avian reproductive performance mainly depend on the hypothalamic-pituitary-ovarian axis. Previous studies have reported that stress affects the secretion of LH and FSH in turkey poults [40], laying hens [8,41], and turkey hens [42]. Thus, successful and profitable poultry production requires understanding the negative effects of environmental conditions and overcoming them [43,44]. *M. oleifera* could be used as a valuable feed additive in animal nutrition due to its unique nutritional profile [21]. The *Moringa* seeds contain a high percentage of sweet oil (30%–40% of the seed weight) and contain around 76% of polyunsaturated fatty acids that can control cholesterol [45]. Additionally, the seeds of *Moringa oleifera* are a source of protein, iron, calcium, ascorbic acid vitamin A, and antioxidant compounds, such as carotenoids, flavonoids, vitamin E, and phenolics [46]. The presence of vitamins and minerals benefit the immune system and cure a myriad of diseases [19,43,44,47,48].

Moreover, *M. oleifera* has been considered a dietary treatment option for heat stress in different animal species [37,38,40,42,49]. In the present study, *M. oleifera* seed powder supplementation influenced the expressions of *ESR2*, *FSHR*, *STAR*, and *PRLR* genes (Figure 1). Compared with the basal diet alone, basal diet supplemented with a 0.3% *M. oleifera* seed powder significantly increased the mRNA expressions of *FSHR*, *ESR2*, and *STAR* in the ovaries of laying quail. This effect was associated with the action of 0.3% *M. oleifera* seed powder in promoting the secretion of reproductive hormones, which could subsequently enhance the mRNA expressions of genes related to the reproductive axis. The activity of FSH and its receptors affect the development of reproductive organs as well as ovulation in follicles [50]. In the present study, dietary supplementation with 0.3% *M. oleifera* seed powder was considered ideal for improving the laying performance (egg weight and egg production) of quail subjected to heat stress (Table 5). Similarly, Riry et al. [51] and Ahmad et al. [38] reported the positive effects of *M. oleifera* seed meal on egg weight and egg production in laying quail and hens, respectively. Other studies have demonstrated the important role of *ESR2* in the maintenance of ovarian function and effective reproduction, and a close relationship between *ESR2* and laying performance was suggested [14,52]. Furthermore, Rangel et al. [53] suggested that *STAR* plays a vital role in the circadian control of steroidogenesis in the F1 follicle and subsequently in the circadian timing of ovulation. Based on these findings of previous studies and that of the present study regarding significant increases in egg production in the T3 group, we suggest that 0.3% *M. oleifera* seed powder supplementation in quail diet increases the expressions of some ovarian genes, thereby, improving the laying performance of quail. Conversely, the expressions of selected genes (*ESR2* and *FSHR*) decreased in the T1 and T2 groups, possibly due to heat stress. Li et al. [11] have suggested that heat stress induces granulosa cell apoptosis and reduces steroidogenic gene mRNA expressions. Previous studies on mammals have demonstrated that heat stress inhibits the development of ovarian follicles and tends to cause ovarian regression [54,55].

In the present study, the results concerning egg quality showed a consistent trend, and the best traits were observed in the T3 group. Riry et al. [51] observed that dietary supplementation with *M. oleifera* seed meal at 5% and 7.5%, significantly improved the albumen (%), yolk (%), and the egg yolk index in laying quail. Furthermore, Abdel-Azeem et al. [56] recorded a high albumen weight, yolk weight, and shell thickness in laying chickens fed a diet supplemented with 0.5%, 1.5%, and 2% *M. oleifera* leaf powder. We could suggest that *M. oleifera* seed powder is beneficial as a dietary supplement in Japanese laying quail for improving productive performance, under heat stress.

## 5. Conclusions

Using *M. oleifera* seed powder as feed additives in Japanese laying quail diets could be a useful strategy for preventing the harmful effects of heat stress on performance and reproductive egg traits. The present study revealed that feed diets supplemented with 0.3% *M. oleifera* seed powder during the laying period, significantly enhanced egg productivity as well as egg quality traits, under heat stress rearing conditions. Furthermore, the expressions of some ovarian genes significantly improved in correlation to fortified feed diets with 0.2% or 0.3% *M. oleifera* seed powder.

## Figures and Tables

**Table 1:** Composition and nutrient content (per 100 g plant) of the *Moringa oleifera* seeds powder.

Parameter	Amount
Moisture (g)	8
Crude protein (g)	36
Crude fat(g)	37.2
Carbohydrate (g)	7.9
Fiber (g)	3.1
Ash (g)	14.2
Calcium (mg)	45
Magnesium (mg)	598
Phosphorus (mg)	73
Potassium (mg)	7
Copper (mg)	4.2
Iron (mg)	2
Sulfur (mg)	0.05
Vitamin E (mg)	766
Vitamin C (mg)	6.5
Total amino acids(g)	20.2
PUFA (mg)	73

Values in the table are those provided by the manufacturer (Agricultural Research Center, Giza, Egypt). PUFA—polyunsaturated fatty acids (linoleic, linolenic, and oleic acid).

**Table 2:** Composition and nutrient level of the basal diet on a dry matter basis.

Items	Amount
Ingredients, %	
Yellow corn	56
Soybean meal (44%)	29.29
Corn gluten meal	3.8
Vegetable oil	3.5
Salt	0.31
Dicalcium phosphate ^1^	1.1
Premix ^2^	0.25
Limestone	5.6
DL-Methionine ^3^	0.11
L-lysine	0.04
Total	100.00
Energy and Nutrient level	
ME (Kcal /kg)	2979
Crude protein %	19.9
Ether extract %	5.9
Crude fiber %	3.3
Calcium %	2.461
Available phosphorus%	0.33
Methionine %	0.454
Lysine	1.002
Methionine + cysteine	0.788

^1^ Dicalcium phosphate, 18% granular phosphate, and 23% calcium. ^2^ Supplied per kilogram of diet: Vitamin A 12,000 IU, vitamin D3 3000 IU, vitamin E 40 mg, vitamin K3 3 mg, vitamin B1 2 mg, vitamin B2 6 mg, vitamin B6 5 mg, vitamin B12 0.02 mg, niacin 45 mg, biotin 0.075 mg, folic acid 2 mg, pantothenic acid 12 mg, manganese 100 mg, zinc 600 mg, iron 30 mg, copper 10 mg, iodine 1 mg, selenium 0.2 mg, and cobalt 0.1 mg. ^3^ DL-Methionine, Met AMINO^®^ (DL-2-amino-4-(methyl-thio)-butane acid, DL-methionine, α-amino-Y-methyl-oily acid) by Feed Grade 99% (EU).

**Table 3:** Used primers for the quantitative real-time PCR.

Product Length (bp)	Primer Sequence (5′–3′)	Gene and ID
*ESR2* (XM_015865466.1)	F: TCACCTGCTGGTATTGGCTC R: GGGATGTAGACTGGACTGTGT	83
*FSHR* (XM_015856889.1)	F: CTCTCGGTCTACACGCTGAC R: TTGCGGTTAAGTTGCATGGC	77
*STAR* (XM_015883089.1)	F: ATCCCAGCGTCAAAGAGGTG R: CCCAATGATGTTCCCAGGCA	98
*PRLR* (NM_001323231.1)	F: AAATGCTGGCGTGAGGTACA R: CCACTGGGGATCAGAATCCG	99
*ß-actin* (NM-205518.1)	F: ATGGCTCCGGTATGTGCAA R: TGTCTTTCTGGCCCATACCAA	120

*ESR2* = Estrogen receptor 2 (ER beta)*, FSHR* = Follicle stimulating hormone receptor, *STAR* = Steroidogenic acute regulatory protein, *PRLR* = Prolactin receptor, and ß-actin = Beta actin.

**Table 4:** Effect of the *Moringa oleifera* supplementation on the laying performance of Japanese quail, during the experimental period.

Item ^1^	Dietary Treatments ^2^					Sig.
	Weeks	CNT	T1	T2	T3	
Egg weight (g/egg)	7^th^–11^th^	11.41 ± 0.25	11.66 ± 0.25	11.73 ± 0.26	11.55 ± 0.25	N.S.
	11^th^-15^th^	11.27 ± 0.27 ^c^	12.07 ± 0.27 ^b^	12.91 ± 0.27 ^a^	12.29 ± 0.28 ^ab^	**
Egg production (%)	7^th^–11^th^	50.51 ± 1.81 ^c^	52.08 ± 1.76 ^c^	59.37 ± 0.76 ^b^	62.20 ± 1.44 ^a^	**
	11^th^–15^th^	48.38 ± 1.95 ^c^	58.97 ± 1.87 ^b^	57.52 ± 1.11 ^b^	65.06 ± 1.91 ^a^	***
Feed intake (g/bird)	7^th^–11^th^	34.75 ± 0.85	33.75 ± 0.63	34.50 ± 1.71	32.00 ± 1.63	N.S.
	11^th^–15^th^	33.75 ± 0.25 ^b^	38.50 ± 1.19 ^a^	29.75 ± 1.25 ^c^	30.75 ± 0.63 ^c^	*
Feed conversion ratio	7^th^–11^th^	3.08 ± 0.10 ^a^	2.65 ± 0.18 ^b^	2.80 ± 0.20 ^b^	2.70 ± 0.17 ^b^	*
	11^th^–15^th^	3.11 ± 0.09 ^a^	2.81 ± 0.10 ^b^	2.49 ± 0.09 ^c^	2.47 ± 0.99 ^c^	**

^1^ Feed conversion ratio = feed intake (g)/egg mass (g); Egg production = 100 [number of eggs laid ÷ (number of hens x number of days)]. ^2^ CNT, Control group; T1, 0.1% *Moringa oleifera* seed powder; T2, 0.2% *Moringa oleifera* seed powder; T3, 0.3% *Moringa oleifera* seed powder. ^a,b,c^ values in the same row with a different superscript differ significantly at *p* ˂ 0.05. Sig.—significance; N.S.—non-significance; * *p* < 0.05, ** *p* < 0.01, *** *p* < 0.001.

**Table 5:** Effect of the *Moringa oleifera* supplementation on egg quality traits, in the 4th and 8th week of the experimental period.

Item	Dietary Treatments ^1^					Sig.
	Week	CNT	T1	T2	T3	
Shell Thickness (mm)	4^th^	0.36 ± 0.22	0.38 ± 0.15	0.42 ± 0.20	0.33 ± 0.23	N.S.
	8^th^	0.30 ± 0.2 ^b^	0.39 ± 0.1 ^a^	0.42 ± 0.25 ^a^	0.39 ± 0.17 ^a^	**
Shell%	4^th^	15.27 ± 0.40	14.26 ± 0.50	15.34 ± 0.5	14.08 ± 0.46	N.S.
	8^th^	14.63 ± 0.55 ^b^	14.27 ± 0.45 ^b^	16.91 ± 0.35 ^a^	14.25 ± 0.55 ^b^	***
Albumin%	4^th^	46.49 ± 2.02	47.91 ± 2.27	50.77 ± 1.75	49.84 ± 2.02	N.S.
	8^th^	54.88 ± 2.50 ^b^	54.17 ± 2.48 ^b^	55.05 ± 2.05 ^b^	57.46 ± 1.99 ^a^	**
Yolk %	4^th^	30.38 ± 0.91 ^b^	32.06 ± 0.97 ^a^	33.30 ± 0.74 ^a^	32.88 ± 0.85 ^a^	*
	8^th^	29.30 ± 1.40 ^b^	30.31 ± 1.61 ^ab^	31.97 ± 1.40 ^a^	30.11 ± 0.99 ^ab^	**
Yolk Index	4^th^	0.32 ± 0.012	0.32 ± 0.014	0.32 ± 0.015	0.32 ± 0.010	N.S.
	8^th^	0.38 ± 0.012 ^b^	0.39 ± 0.009 ^b^	0.38 ± 0.013 ^b^	0.45 ± 0.015 ^a^	**
Egg Shape Index	4^th^	77.20 ± 0.81	77.75 ± 0.82	77.50 ± 0.78	78.52 ± 0.80	N.S.
	8^th^	77.40 ± 0.77	76.75 ± 0.92	78.45 ± 0.90	79.05 ± 0.80	N.S.
Haugh Unit	4^th^	83.08 ± 1.18	84.61 ± 1.16	83.45 ± 1.07	82.50 ± 0.98	N.S.
	8^th^	81.18 ± 1.32	81.14 ± 1.27	81.54 ± 1.19	82.31 ± 1.22	N.S.

^1^ CNT, Control group; T1, 0.1% *Moringa oleifera* seed powder; T2, 0.2% *Moringa oleifera* seed powder; T3, 0.3% *Moringa oleifera* seed powder. ^a,b^ values in the same row with a different superscript differ significantly at *p* < 0.05. Sig.—significance; N.S.—non-significance; * *p* < 0.05, ** *p* < 0.01, *** *p* < 0.001.
